# Factors associated with poor physical performance in older adults of 11 Peruvian high Andean communities

**DOI:** 10.12688/f1000research.17513.2

**Published:** 2019-09-10

**Authors:** Diego Urrunaga-Pastor, Fernando M. Runzer-Colmenares, Tania M. Arones, Rosario Meza-Cordero, Silvana Taipe-Guizado, Jack M. Guralnik, Jose F. Parodi

**Affiliations:** 1 Unidad de Investigación para la Generación y Síntesis de Evidencias en Salud, Universidad San Ignacio de Loyola, Lima, 15024, Peru; 2 Universidad de San Martín de Porres, Facultad de Medicina Humana, Centro de Investigación del Envejecimiento (CIEN), Lima, Peru; 3 Bamboo Seniors Health Services, Lima, 15038, Peru; 4 Universidad Científica del Sur, Facultad de Ciencias de la Salud, Carrera de Medicina Humana, Lima, 15067, Peru; 5 Sociedad Científica de Estudiantes de Medicina de la Universidad de San Martín de Porres, Universidad de San Martín de Porres, Lima, 15024, Peru; 6 University of Maryland, School of Medicine, Baltimore, Maryland, 21211, USA

**Keywords:** Physical performance, Altitude, Elderly, Latin America, Peru

## Abstract

**Background:** Physical performance in the older adult has been extensively studied. However, only a few studies have evaluated physical performance among older adults of high Andean populations and none have studied the factors associated with it. The objective of this study was to evaluate factors associated with poor physical performance by using the Short Physical Performance Battery (SPPB) in older adults living in 11 Peruvian high Andean communities.

**Methods: **An analytical cross-sectional study was carried out in inhabitants aged 60 or over from 11 high-altitude Andean communities of Peru during 2013-2017. Participants were categorized in two groups according to their SPPB score: poor physical performance (0-6 points) and medium/good physical performance (7-12 points). Additionally, we collected socio-demographic, medical, functional and cognitive assessment information. Poisson regression models were constructed to identify factors associated with poor physical performance. Prevalence ratio (PR) with 95% confidence intervals (95 CI%) are presented.

**Results: **A total of 407 older adults were studied. The average age was 73.0 ± 6.9 years (range: 60-94 years) and 181 (44.5%) participants had poor physical performance (0-6 points). In the adjusted Poisson regression analysis, the factors associated with poor physical performance were: female gender (PR=1.29; 95%CI: 1.03-1.61), lack of social support (PR=2.10; 95%CI: 1.17-3.76), number of drugs used (PR=1.09; 95%CI: 1.01-1.17), urinary incontinence (PR=1.45; 95%CI: 1.16-1.82), exhaustion (PR=1.35; 95%CI: 1.03-1.75) and cognitive impairment (PR=1.89; 95%CI: 1.40-2.55).

**Conclusions:** Almost half of the population evaluated had poor physical performance based on the SPPB. Factors that would increase the possibility of suffering from poor physical performance were: female gender, lack of social support, number of drugs used, urinary incontinence, exhaustion and cognitive impairment. Future studies with a larger sample and longitudinal follow-up are needed to design beneficial interventions for the high Andean population.

## Introduction

Aging is a physiological process that involves changes in respiratory, cardiovascular, muscular, kidney and brain function ^1– 4^. In addition, these changes organically could be exacerbated in older adults living at high altitude due to the hypoxia to which they are chronically exposed, increasing their risk of suffering certain pathologies; however, there is no consensus surrounding this situation ^5, 6^. Chronic mountain sickness is a clinical syndrome that affects natives or residents living for a long time at an altitude greater than 2500 meters above sea level (masl) and is characterized by erythrocytosis that could evolve to severe pulmonary hypertension and generate congestive heart failure, affecting the ability of Andean older adults to maintain their daily activities and their physical performance ^7^.

Physical performance in the older adult has been extensively studied, and poor nutritional status ^8^, sarcopenia ^9^, decreased muscle mass, frailty ^10^, sarcopenic obesity ^11^, mortality ^12^, disability ^13^ and dementia ^14^, common chronic diseases of aging, have been associated. A previous study conducted in rural Peruvian communities located at 3345 and 6 masl found that the prevalence of poor physical performance in older adults living in rural communities at sea level was twice as high as that of older adults that are residing in rural areas at high altitude ^15^.

Previous studies in high-altitude communities have described older population´s nutritional status, finding a prevalence rates of 9.4% for malnutrition ^16^, 17.6% for sarcopenia ^17^, 15.2% for insomnia ^18^, 12.2% for frailty ^19^ and 75.2% for fear of falling ^19^. These figures are similar to those described in populations at sea level ^20– 23^. At high altitudes, an increased ventilatory response and a lower cardiac response to hypoxia will favor oxygen uptake in the lungs and allow the maintenance of a normal oxygen saturation, at moderate exercise ^24^. In addition, tissue hypoxia, oxidative stress and the action of free radicals would be increased, affecting cardiac energy metabolism and skeletal muscle performance; in this way, a decrease in mitochondrial volume would be generated. This situation would occur in people exposed for a long time or who have returned from high altitude ^25, 26^ and significantly affect the physical performance of the older adult living at high altitude; however, there is no clear consensus regarding this process ^26– 28^, especially in the elderly.

Additionally, there are no parameters or determinants in relation to the poor physical performance in older adults of the Andes, which could be different from those described for other populations, due to social or geographical conditions, or due to access to health services. Therefore, this study aimed to determine the factors associated with poor physical performance in older adults from 11 high Andean communities in Peru.

## Methods

### Design and population

Analytical cross-sectional study, carried out in inhabitants aged 60 or over from 11 high-altitude (≥1500 masl) ^29^ Andean communities of Peru: La Jalca, Leimebamba (Amazonas), Llupa, San Pedro de Chaná, Atipayan (Áncash), Pampamarca (Huánuco), Chacapampa (Huancayo), Ayahuanco (Ayacucho), Paucarcolla (Puno), Vilca (Huancavelica) and Viñac (Lima) during 2013–2017 period. All inhabitants of the 11 high-altitude Andean communities included, belonged to the same ethnic group and performed a similar work activity, based mainly on agriculture, farming and trading ^30^.

### Description of the study area

The National Statistics Institute of Peru (Instituto Nacional de Estadística e Informática -INEI) classifies communities with 100 houses not in a capital district, that have more than 100 individuals, located in a dispersed way without forming blocks as rural communities ^31^. The communities were located in the Peruvian highlands as follows: a) *La Jalca*: urban settlement located at 2800 masl; b) *Leimebamba*: rural village located at 2158 masl; c) *Llupa*: rural village located at 3511 masl; d) *San Pedro de Chaná*: rural village located at 3413 masl; e) *Atipayán*: rural village located at 3364 masl; f) *Pampamarca*: urban village located at 3445 masl; g) *Chacapampa*: rural village located at 3358 masl; h) *Ayahuanco*: rural village located at 3414 masl; i) *Paucarcolla*: urban village located at 3847 masl; j) *Vilca*: rural village located at 3275 masl; k) *Viñac*: rural village located at 3315 masl. The Peruvian Andes weather biodiversity includes high temperatures, rainfall and cloudy seasons ^32^. These areas have low levels of pollution; however, mining activities are endangering ecosystems sustainability ^33^.

### Sample type, sample size and analysis unit

A non-probabilistic, census-type sampling was performed, registering all the elderly people in the highland communities previously described. We included all or most (approximately 95%) of the geriatric population of each community (urban/rural) ^34^. The analysis unit was elderly person from high-altitude Andean communities (rural/urban). The final sample included 413 older adults who voluntarily signed an informed consent form accepting their participation in the study.

### Evaluation

Participants were visited in their homes up to three times to be invited to participate in the study. Those who agreed to participate voluntarily signed a document of informed consent prior to the collection of data by the researchers ^34^. Data was collected on sociodemographic characteristics, medical background (falls, polypharmacy, comorbidities, tobacco, alcohol and coca leaf consumption), Barthel Index, Edmonton test, exhaustion) ^35– 37^, physical performance (Short Physical Performance Battery) ^12^, anthropometric measurements (height and weight) and cognitive status (Yesavage test and Pfeiffer Questionnaire) ^38, 39^. The interview was conducted by a geriatrician, medical doctors and medical students (previously trained by the geriatrician). All the self-reported data was collected during the interview and in case the participants did not speak Spanish, the questionnaires were translated by the caregiver/family member of the participant at the time of data collection.

### Measures

***Outcome: Poor physical performance.*** To evaluate physical performance in the participants, we used the Short Physical Performance Battery (SPPB). The SPPB is based on three timed tasks: standing balance, walking or gait speed, and five repetitive chair stands. The timed results of each subtest are rescaled according to predefined cut points for obtaining a score ranging from 0 (worst performance) to 12 (best performance) ^40^. The variable was categorized as: poor physical performance (0-6) and medium/good physical performance (7-12) ^12, 15^.

***Other variables***

*Sociodemographic characteristics.* The sociodemographic characteristics included and evaluated by self-report were: age (less than or equal to 70 years, 71 to 80 years, over 80 years), gender (male, female), educational level (no education/incomplete elemental school, complete elemental school, complete high school), marital status (single, married, widowed/divorced), live alone (yes or no), time by foot from their home to the nearest health centre (in minutes) and altitude (masl). The sociodemographic information was corroborated with the participant's national identity document (ID card).

*Medical background.* The following variables were included and evaluated by self-report: falls in the last year (none, at least 1), hospitalizations in the last year (none, at least 1), polypharmacy (5 drugs or more, under medical prescription) ^41^, tobacco consumption (yes or no), alcohol consumption (yes or no), coca leaf consumption (yes or no), high blood pressure (HBP) (yes or no), diabetes mellitus type 2 (DM2) (yes or no), chronic obstructive pulmonary disease (COPD) (yes or no) and low back pain (yes or no). Likewise, a variable of comorbidities (obesity defined according to body mass index (BMI) + HBP + COPD + DM2 + low back pain) was constructed ^34, 42^. The medical background information was confirmed by the caregiver/family member at the time of data collection.

We determined the body mass index (BMI), which was calculated with the formula weight in kg/(size in meters squared). This was categorized as follows: malnutrition (<18.5 kg/m ^2^), normal (18.5-24.99 kg/m ^2^), overweight (25.0-29.99 kg/m ^2^) and obesity (>30.0 kg/m ^2^) ^43^.

*Functional assessment.* We used the Barthel Index, a questionnaire about 10 basic activities of daily living (ADL) with a total score between 0–100. It was analyzed as a continuous variable and also divided into two strata: independent (100) and dependent (<100) ^35, 44^.

Additionally, we use two items from the Edmonton test: 1) social support: When you need help, do you have someone who meets your needs? (always, sometimes/never); 2) urinary incontinence: Do you have trouble holding urine when you do not feel like urinating? (yes or no). The Edmonton test has 9 items and is used to evaluate frailty ^36^.

In the present study, we evaluated exhaustion, which is defined by 3 items that the participant must respond according to the way he felt during the last 2 weeks: 1) did you feel full of energy? (yes or no); 2) did you feel that you could not go on? (yes or no); 3) did you feel that all you did was with effort? (yes or no). A score equal or greater than two was considered positive for exhaustion dimension ^37, 45^.

*Psychological and cognitive assessment.* We used the Yesavage test, which is a 5-item questionnaire that evaluates the presence of depressive symptoms. A score equal or greater than three was considered positive for depressive symptoms ^38^.

We used the Pfeiffer Questionnaire, a 10-item questionnaire for evaluation of cognitive impairment. The strata were generated as follows: no impairment (0 to 2 errors), mild impairment (3 to 4 errors), moderate impairment (5-7 errors) ^39^.

### Statistical analysis

We used STATA v14.0 for our analysis. Descriptive results were presented using measures of central tendency, dispersion measures, absolute frequencies, and relative frequencies. The characteristics of the participants with poor and medium/good physical performance were compared using the Chi square test, Fisher’s exact test, Student’s T test or the Wilcoxon rank sum test as appropriate.

Two Poisson regression models (1 crude and 1 adjusted) were constructed using robust variance with the objective of evaluating factors associated with poor physical performance in the participants. We decided to use Poisson regression to avoid overestimating the calculated associations. The reported measure was the prevalence ratio (PR) with their respective 95% confidence intervals (95%CI).

The adjusted model included the following variables: gender, lack of social support, alcohol consumption, tobacco consumption, number of drugs used, comorbidities, urinary incontinence, falls in the last year, hospitalizations in the last year, dependence ADL, exhaustion, depressive symptoms, exhaustion, cognitive impairment and altitude (masl). These variables were included in the adjusted model because they had statistically significant association with poor physical performance in the crude Poisson regression analysis. Additionally, we evaluated the possible collinearity between the exposure variables entered in the adjusted model and the study data complied with the statistical assumptions of the Poisson regression.

### Ethical issues

The research project was approved by the Institutional Review Board of the Peruvian Naval Medical Centre, located in Lima, Peru. Informed consent was obtained from all the participants. In case of cognitive impairment, the family member who was present at the time of data collection gave the written consent. Furthermore, the anonymity of the participants and confidentiality of the data were ensured.

## Results

### Sociodemographic characteristics of the study sample and bivariate analysis

Of a total of 413 elderly adults, 3 participants were excluded because of severe cognitive impairment, equivalent to a score equal or greater than 8 in the Pfeiffer Questionnaire, 2 participants were excluded because they did not have variables of interest and 1 participant was excluded because of being physically incapable of performing the physical and functional performance tests (visual and auditory impairment). Finally, a total of 407 individuals were analyzed.

Data from 407 elderly adults from 11 high Andean communities were analyzed. In total, 181 (44.5%) participants had poor physical performance and the SPPB mean was 7.3 ± 3.1. The mean age was 73.0 ± 6.9 years old (range: 60–94 years old), 267 (65.6%) participants were female, 335 (82.3%) did not count with education or had not finished elementary school, 271 (77.2%) worked in agriculture and 91 (22.4%) lived alone. Statistically significant differences were found in gender, educational level, live alone, time by foot from their home to the nearest health centre (in minutes) and altitude (masl) among physical performance groups ( Table 1). Full raw data are available on OSF ^46^.

**Table 1.** Sociodemographic characteristics of the study sample and bivariate analysis.

			Physical performance		
Variables	N	%	Medium/ Good, n (%)	Poor, n (%)	P value
Total	407	100	226 (55.5)	181 (44.5)	
Gender					<0.001
Female	267	65.6	131 (49.1)	136 (50.9)	
Male	140	34.4	95 (67.9)	45 (32.1)	
Age *	73.0 ± 6.9		72.5 ± 6.8	73.6 ± 7.1	0.133
≤70 years	167	41.0	98 (58.7)	69 (41.3)	0.181
71–80 years	176	43.3	99 (56.3)	77 (43.7)	
>80 years	64	15.7	29 (45.3)	35 (54.7)	
Marital status					0.680
Single	42	10.3	26 (61.9)	16 (38.1)	
Married	237	58.2	130 (54.9)	107 (45.1)	
Widowed/divorced	128	31.5	70 (54.7)	58 (45.3)	
Educational level					0.003
No education/Incomplete elemental school	335	82.3	174 (51.9)	161 (48.1)	
Complete elemental school	70	17.2	50 (71.4)	20 (28.6)	
Complete high school	2	0.5	2 (100.0)	0 (0.0)	
Live alone					
Yes	91	22.4	62 (68.1)	29 (31.9)	0.006
Time by foot from their home to the nearest health center (in minutes) **	15 (10-30)		15 (10-30)	20 (15-25)	0.026
Altitude (masl) **	3414 (3275-3511)	3364 (3275-3445)	3414 (3315-3511)		<0.001

*Mean ± standard deviation. **Median (interquartile range).

### Medical background, functional, psychological and cognitive tests in the study sample and bivariate analysis

Of the 407 elderly adults evaluated, 261 (64.3%) had at least 1 fall in the last year, 48 (11.8%) were hospitalized at least once in the last year, 74 (18.2%) consumed coca leaf, 109 (19.4%) were obese according to BMI, 337 (83.0%) had disability (Barthel Index), 150 (36.9%) had depressive symptoms and 116 (28.5%) had cognitive impairment (mild-moderate) ( Table 2).

**Table 2.** Medical background, functional assessment and cognitive evaluation in the study sample and bivariate analysis.

			Physical performance		
Variables	N	%	Medium/Good, n (%)	Poor, n (%)	P value
**Total**	407	100	226 (55.5)	181 (44.5)	
**Medical background**					
Falls in the last year					<0.001
None	145	35.7	117 (80.7)	28 (19.3)	
At least 1	261	64.3	108 (41.4)	153 (58.6)	
Hospitalizations					0.041
None	358	88.2	205 (57.3)	153 (42.7)	
At least 1	48	11.8	20 (41.7)	28 (58.3)	
Tobacco consumption					<0.001
Yes	49	12.0	15 (30.6)	34 (69.4)	
Alcohol consumption					<0.001
Yes	116	28.5	41 (35.3)	75 (64.7)	
Coca leaf consumption					0.981
Yes	74	18.2	41 (55.4)	33 (44.6)	
Number of drugs used *	1 (0-2)		0 (0-1)	1 (0-3)	<0.001
Polypharmacy					<0.001
Yes	13	3.2	1 (7.7)	12 (92.3)	
Comorbidities *	0 (0-1)		0 (0-1)	1 (0-1)	0.076
HBP	44	10.8	23 (52.3)	21 (47.7)	0.645
COPD	16	3.9	10 (62.5)	6 (37.5)	0.567
DM2	31	7.6	16 (51.6)	15 (48.4)	0.648
Low back pain	75	18.4	43 (57.3)	32 (42.7)	0.728
BMI					<0.001
Malnutrition	3	0.7	2 (66.7)	1 (33.3)	
Normal	162	39.8	108 (66.7)	54 (33.3)	
Overweight	133	32.7	73 (54.9)	60 (45.1)	
Obesity	109	26.8	43 (39.5)	66 (60.5)	
**Functional assessment**					
Barthel Index *	0 (0-95)		70 (0-95)	0 (0-95)	0.021
Independent	69	17.0	48 (69.6)	21 (30.4)	0.011
Dependent	337	83.0	178 (52.8)	159 (47.2)	
Social support					<0.001
Always	182	45.1	134 (73.6)	48 (26.4)	
Sometimes/never	222	54.9	89 (40.1)	133 (59.9)	
Urinary incontinence					<0.001
Positive	116	32.1	42 (36.2)	74 (63.8)	
Exhaustion					<0.001
Positive	156	45.2	72 (46.2)	84 (53.8)	
**Psychological and Cognitive Assessment**					
Depressive symptoms					<0.001
Positive	150	36.9	62 (41.3)	88 (58.7)	
Pffeifer Questionnaire					<0.001
No impairment	291	71.5	200 (68.7)	91 (31.3)	
Mild impairment	100	24.6	22 (22.0)	78 (78.0)	
Moderate impairment	16	3.9	4 (25.0)	12 (75.0)	

*Median (interquartile range). HBP, high blood pressure; COPD, chronic obstructive pulmonary disease. DM2, diabetes mellitus type 2; BMI, body mass index.

### Factors associated with poor physical performance

In the adjusted Poisson regression analysis, the factors associated with poor physical performance were: female gender (PR=1.29; 95%CI: 1.03-1.61), lack of social support (PR=2.10; 95%CI: 1.17-3.76), number of drugs used (PR=1.09; 95%CI: 1.01-1.17), urinary incontinence (PR=1.45; 95%CI: 1.16-1.82), exhaustion (PR=1.35; 95%CI: 1.03-1.75) and cognitive impairment (PR=1.89; 95%CI: 1.40-2.55) ( Table 3).

**Table 3.** Poisson regression to determine factors associated with poor physical performance.

Variables	Crude Model: PR (95%IC)	P value	Adjusted Model: PR (95%IC)	P value
Female gender	**1.58 (1.21-2.07)**	**0.001**	**1.29 (1.03-1.61)**	**0.028**
Lack of social support	**2.27 (1.74-2.96)**	**<0.001**	**2.10 (1.17-3.76)**	**0.013**
Alcohol consumption	**1.77 (1.45-2.17)**	**<0.001**	0.95 (0.77-1.19)	0.673
Tobacco consumption	**1.69 (1.35-2.11)**	**<0.001**	0.95 (0.74-1.22)	0.694
Number of drugs used	**1.22 (1.17-1.27)**	**<0.001**	**1.09 (1.01-1.17)**	**0.022**
Comorbidities	**1.15 (1.02-1.29)**	**0.019**	0.95 (0.86-1.06)	0.364
Urinary incontinence	**2.08 (1.65-2.63)**	**<0.001**	**1.45 (1.16-1.82)**	**0.001**
Falls in the last year	**3.04 (2.14-4.30)**	**<0.001**	1.57 (0.87-2.83)	0.134
Hospitalizations in the last year	**1.36 (1.04-1.78)**	**0.023**	0.98 (0.74-1.29)	0.877
Dependence ADL ^1^	**1.55 (1.07-2.25)**	**0.022**	2.03 (0.94-4.39)	0.071
Exhaustion	**2.04 (1.54-2.69)**	**<0.001**	**1.35 (1.03-1.75)**	**0.027**
Depressive symptoms	**1.62 (1.31-2.00)**	**<0.001**	1.26 (0.98-1.62)	0.072
Cognitive impairment (Pffeifer Questionnaire score ≥3)	**2.48 (2.04-3.02)**	**<0.001**	**1.89 (1.40-2.55)**	**<0.001**
Altitude (masl) ^2^	**1.47 (1.11-1.95)**	**0.007**	0.86 (0.73-1.02)	0.089

^1^Activities of daily living, assessed with Barthel Index. ^2^Altitude for each 1000 masl.

## Discussion

A total of 407 older adults from 11 high Andean communities were analyzed, of whom 44.5% had poor physical performance. Factors that would increase the possibility of suffering poor physical performance were: female gender, lack of social support, number of drugs used, urinary incontinence, exhaustion and cognitive impairment.

Previous studies evaluated physical performance using SPPB as a measurement tool, finding diverse results. One of them, conducted in the United States with 631 older adults, calculated a SPPB average score of 9.9 in its participants, being higher than the 7.3 points found as a mean score in our population. However, the sample size of older adults lived in urban areas and was higher than the one we assessed ^47^. On the other hand, in the InCHIANTI study cohort conducted in 542 older adults from Italy, it was found that approximately 65% of the participants with a SPPB score less than or equal to 7 were unable to complete the 400 meters walk test after the three years of follow-up, being a higher proportion than the found in our study. In addition, this SPPB score (≤7) was associated with an odds ratio (OR) of approximately 27 predicting inability to complete 400 meters walk test in those able to walk 400 meters at baseline ^48^. Similarly, another study conducted in Italy found a lower SPPB mean score than the one calculated in our study population; nevertheless, this study was performed in hospitalized patients ^49^. Additionally, a previous study carried out in Peru in the rural communities of Atipayán (3345 masl) and Santa (6 masl), showed a prevalence of poor physical performance of 10.0% and 19.4%, respectively, both lower figures to that found in this study ^15^.

We have found a higher SPPB mean score than that found in other studies, highlighting the fact of being a population living in altitude cities. Nevertheless, these findings can only be interpreted for the altitude ranges evaluated in the present study.

We found an association between female gender and poor physical performance in the evaluated population. Equally, a cohort carried out in 3041 well-functioning white and black men and women, aged 70–79 years, found that men independently of the race had a better physical performance than women (evaluated by the knee extension strength, chair-rise, 6 meters walk time, 400 meters walk time and standing balance test) ^50^. In contrast, Vasunilashorn *et al.* ^48^ did not find differences between physical performance groups and gender. This association could be explained because women usually have less muscle mass than men, and menopause produce an acute decline in strength and muscle mass, compared with the gradual loss of strength by men of similar age ^17, 51^.

In this study, association between lack of social support and poor physical performance was found. A systematic review by Vagetti *et al.* during 2014 that aimed to assess the association between physical activity and quality of life in older adults found a moderate association between social support and physical activity in older adults ^52^. Similarly, a study in Norway found a consistent correlation between physical activity in older adults and social support, especially regarding family social support rather than friend-related support ^53^. This association would be explained by the close relationship between the deterioration of physical and mental health caused by the lack of social support in older adults, which would negatively affect the control of diseases and the physical performance of this population ^54^.

The presence of chronic diseases and comorbidities are common in the older people, and require pharmacological therapy in the majority of cases in order to manage them properly ^55^. A study conducted in 1123 hospitalized older adults in Italy found that the prevalence of polypharmacy was higher in patients with poor physical performance and grip strength ^56^. Also, the association between consumption of more than five drugs would be associated with the presence of frailty, disability and falls in older adults, which would significantly affect the physical performance of the elderly ^57, 58^. Due to the absence of an adequate health network in high-altitude areas able to properly provide drugs to older people ^59, 60^, the presence of polypharmacy would be significantly lower than that of the older people in urban areas, limiting the consequences in their physical performance.

We found an association between urinary incontinence and poor physical performance in the population that was evaluated. A study conducted in Taiwan by Chiu *et al.* found an association between poor physical performance and the presence of urinary incontinence in older adults ^61^. Similarly, in a cohort study conducted in 328 older Latinos in the United States, the increase in SPPB score at one-year follow-up was associated with a lower incidence of urinary incontinence ^62^.

In this study, an association between exhaustion and poor physical performance was found. Exhaustion and poor physical performance evaluated by SPPB are useful tools in the evaluation of sarcopenia, frailty and disability ^63– 66^. Previous studies reinforce the association found in this study, describing very low SPPB scores in fragile older people compared to non-fragile older people (2.9 vs. 8.5, respectively) ^67^. In our study population, a high prevalence of exhaustion was found, which could be due to the continuous physical effort that these inhabitants perform in their daily activities, which mainly involve agriculture and trading.

We found no association between poor physical performance and disability. As well as SPPB, the functional reach test, both performance-based measure, was not associated with disability assessed by the Barthel Index in older adults of Peruvian high Andean communities ^34^. In addition, we did not find an association between poor physical performance and altitude in the adjusted regression model. Both associations had statistical significance in the crude regression model; however, in the adjusted model, they lost it. A possible explanation for this could be the sample size, because, in the adjusted model, both associations presented a p-value with marginal significance ^68^. Although p-value is a useful parameter to explain a result based on statistical significance, it is not the only one to be taken into account ^69^.

The relevance of our results allows our research team to hypothesize plausible explanations of the presented findings: 1) people with a high number of comorbidities cannot live at highest altitudes, so we do not find a comorbid population in our study; 2) living at that altitude range makes you physically stronger; 3) there is another variable or condition about the people living at high altitude that was missed in our study and that we did not adjust for in the regression models. In regard of these, the Andean older people work from a very young age in tasks that involve physical effort, so this could be an interesting point of the study. It is also important to indicate that in the crude model, altitude (for each 1000 masl) increased the probability of poor physical performance; however, after we adjusted the analysis including medical, functional and cognitive variables, the high altitude became a protective marker for poor physical function. These questions would serve as a basis for future studies.

Moreover, an association between the presence of cognitive impairment and poor physical performance was found. The protective effect of physical activity against the development of some type of dementia or neurocognitive disorder has been previously described in multiple studies ^70– 72^. In rural populations at sea level and in altitude, the prevalence of cognitive disorders is low; this could be attributed to different lifestyles, such as the constant physical activity they have performed throughout their lives ^15^.

This study has some limitations: 1) the sampling conducted was not probabilistic, the results cannot be extrapolated; nevertheless, this study was conducted in 11 communities at different altitudes, and the participants reported fewer comorbidities than persons in hospitals, drawing closer to the rural reality; 2) because of its cross-sectional design, this study does not allow us to evaluate causality between the poor physical performance and the associated factors; yet, we still could identify useful markers for future intervention studies; 3) we used self-report to collect some variables in this study which can generate a recall bias. Nevertheless, this is not the case of our main variable which was performance-based measured ^73^; 4) low educational level of the studied population would affect the accuracy of self-report to collect information on complex diseases ^42^; hence, we corroborated the data of the most common comorbidities with a family member/caregiver of the respondent at the time of the interview; 5) because of their low educational level, it was not possible to assess the amount of alcohol of tobacco consumed by the participants; 6) some variables studied have missing values, though, they did not exceed 20%, allowing its analysis ^74^.

In conclusion, almost half of the population evaluated had poor physical performance based on the SPPB. Factors that would increase the possibility of suffering from poor physical performance were: female gender, lack of social support, number of drugs used, urinary incontinence, exhaustion and cognitive impairment. These markers would be very important to develop future cohort studies which would like to study more specifically some marker found in this study.

## Data availability

The raw data associated with this study are available on OSF. DOI: https://doi.org/10.17605/OSF.IO/RSC7Q ^46^.

Data are available under the terms of the Creative Commons Zero "No rights reserved" data waiver (CC0 1.0 Public domain dedication).
