# Supplementary material for: Factors associated with poor physical performance in older adults of 11 Peruvian high Andean communities
Source: F1000Res. 2019 Sep 10;8:59. Originally published 2019 Jan 15. [Version 2] doi: 10.12688/f1000research.17513.2 (PMC6415321; doi:10.12688/f1000research.17513.2)
Supplement: Peer review history

# Peer review: Reviewer response for version 1

**DOI:** 10.5256/f1000research.19153.r44368

**Published:** 2019-03-12

**Authors:** Mariella Guerra

**Affiliations:** 1 Memory Institute and Related Disorders (IMEDER), Lima, Peru

- Was an interview for the informant to confirm participant data/information?
- Did you find participants who only speak Quechua or a dialect? If so did you translate the questionnaires?
- Differences between urban and rural areas?
- Need to discuss Peruvian research.
- Discussion must be done around community findings.
- You found no association between poor physical performance and disability. Explanations beside statistical results.

I confirm that I have read this submission and believe that I have an appropriate level of expertise to confirm that it is of an acceptable scientific standard.

# Peer review: Reviewer response for version 1

**DOI:** 10.5256/f1000research.19153.r43044

**Published:** 2019-03-12

**Authors:** Tania Tello Rodriguez

**Affiliations:** 1 Gerontology Institute, Cayetano Heredia University, Lima, Peru

**Introduction**

 Older people from high Andean populations, in the majority of the cases, they do a lot of physical activity. However, the social context and the lack of health access services could be a negative factor for healthy ageing.

**It could be important to add something about physical activity in the introduction**.

 A previous study of physical performance in older adults in rural areas in Peru found better physical performance in those who lived at height compared to those who lived at sea level, then probably height is not the cause to have a better or worse physical performance but other factors such as physical activity, multimorbidity, etc.

**Methods**

 The best design for this kind of study is of case and control but the tranversal studies give us relevant information.

 The presence of osteoarthritis of knee and hip and low physical activity previous can impact in the physical performance in this study.

 In older people the BMI is not the best parameter to evaluate malnutrition and the recommended levels are different, as mentioned in this study.

 Describe the inclusion criteria and exclusion ones in detail, they are partially mentioned in the results.

**Results**

 Related to the comorbility takes my attention the low percentage of arterial hypertension found in 10% and the high frequency of functional dependence is 83%. Very different amounts to that reported in previous studies.

**Discussion**

 83% has functional dependence by the Barthel index, so it is a study to the community that is surprising, one of the hypothesis it is the population has high rates of multimorbility but to collect information but self-report the information was not given.

 There are other variables or conditions about the people living at high altitude that was missed in this study and that were not adjusted for in the regression models.

 The population that has cognitive impairment in a mild-moderate way by the test of Pfeiffer (screening test) could have a low score in the physical performance due to they did not understand in an appropriate way the orders to use SPPB; thus, in the context from people with low educational levels.

 The fact that they do not find links between poor physical performance and to live at high altitude support a previous study done ^1^.

I confirm that I have read this submission and believe that I have an appropriate level of expertise to confirm that it is of an acceptable scientific standard.

## References

[1] : [Physical performance of older adults living in rural areas at sea level and at high altitude in Peru]. *Rev Esp Geriatr Gerontol*.50(2) : 10.1016/j.regg.2014.11.001 56-61 10.1016/j.regg.2014.11.001 25500001

# Peer review: Reviewer response for version 1

**DOI:** 10.5256/f1000research.19153.r43042

**Published:** 2019-03-01

**Authors:** Diego Andrés Chavarro-Carvajal

**Affiliations:** 1 Instituto de Envejecimiento, Facultad de Medicina, Pontificia Universidad Javeriana, Bogotá, Colombia

Urrunaga-Pastor and co-authors evaluated the performance of the physical performance of the Short Physical Performance Battery (SPPB) in older adults living in 11 Peruvian high Andean communities. This work has a clear objective; the results are interesting and provide novel information in these regions of Peru with very particular characteristics given the geographical location and the height above sea level.

 I consider statistical analysis is proper to a cross sectional study using Poisson regression and was reported prevalence ratio with their confidence intervals.

I confirm that I have read this submission and believe that I have an appropriate level of expertise to confirm that it is of an acceptable scientific standard.

# Peer review: Reviewer response for version 1

**DOI:** 10.5256/f1000research.19153.r43043

**Published:** 2019-02-21

**Authors:** Oscar Rosas-Carrasco

**Affiliations:** 1 National Geriatrics Institute, National Institutes of Health, Mexico City, Mexico

This work has a clear objective. The lack of studies in communities far from the big cities will always be a solid point to support the publication of these studies. The strength of the study is that it includes the possibility of studying the physical performance in populations with height above sea level greater than 2500 meters.

 I have some considerations to correct in the methodology:

- I understand that the authors preferred to use a Poisson regression due to the fact that the characteristics of the dependent variable (physical performance) were adequate (by distribution?) to prefer this analysis regression, however it should be specifically noted and included why they did not use logistic regression if the dependent variable presented only had two categories.
- On the other hand, include if medical doctors and medical students were compared with any statistical test to corroborate concordance.
- Do the authors have information about the migration of those with high comorbidity? The above could explain why a low frequency of chronic diseases and good performance were found.
- Include some result of the final Poisson regression model that allows to know if the fit of model regression were adequate to present the results obtained.

In conclusion, the manuscript must be accepted with some corrections that the authors must consider, the results are interesting and provide novel information in these regions of Peru.

I confirm that I have read this submission and believe that I have an appropriate level of expertise to confirm that it is of an acceptable scientific standard, however I have significant reservations, as outlined above.
